# Measuring Kinematic Viscosity of Engine Oils: A Comparison of Data Obtained from Four Different Devices

**DOI:** 10.3390/s21072530

**Published:** 2021-04-04

**Authors:** Artur Wolak, Grzegorz Zając, Tomasz Słowik

**Affiliations:** 1 Department of Quality and Safety of Industrial Products, Cracow University of Economics, Rakowicka 27, 31-510 Kraków, Poland; artur.wolak@uek.krakow.pl; 2 Department of Power Engineering and Transportation, Faculty of Production Engineering, University of Life Sciences in Lublin, Głęboka 28, 20-612 Lublin, Poland; tomasz.slowik@up.lublin.pl

**Keywords:** lubricant properties, degradation, engine oil, viscosity, mid-FTIR, Stabinger viscometer, microchannel viscometer, Ubbelohde Capillary viscometer, reliability of results

## Abstract

The aim of this paper is to compare the results of kinematic viscosity of lubricating oils measurements at 40 °C, obtained with three different rapid evaluation devices, and the standardized method using an Ubbelohde Capillary viscometer. The following instruments were selected to measure: a mid-FTIR spectrophotometer, a microchannel viscometer, and a Stabinger viscometer. The study material comprised 42 fresh engine oils, all of which are commercially available. The main data analysis tools used in the study were multiple regression, Mahala Nobis distance, post-hoc analysis, and the Wilcoxon signed-rank test with the Bonferroni correction. Consistent outcomes were obtained for the Stabinger viscometer only, whereas the microchannel viscometer and the mid-FTIR spectrophotometer were not as precise as the reference method. It was also found that the results obtained with the use of the mid-FTIR spectrophotometer were burdened with a very large measurement error. Therefore, a very careful approach is suggested when choosing these instruments. The study fills an important gap in empirical research in the context of the reliability of measurement results obtained using various research techniques.

## 1. Introduction

Engine oil ensures smooth engine operation through its lubricating and cooling effects; yet, with time and use, it becomes subject to degradation processes. High temperature; solid impurities (such as soot, coke, products of tribological wear of the engine, corrosion products, etc.); and the external ones (moisture, air dust, air, and other gases), as well as shear, oxidation, and nitration processes are some of the key factors contributing to the degradation of engine oil [1]. It is then particularly important to monitor the properties of oil; especially, as its degradation can eventually lead to engine damage or failure [2]. In fact, in the automotive industry, monitoring the viscosity of lubricants is considered a principal indicator of oil quality [3,4]. Viscosity values may increase or decrease during operation [5]. An increase in kinematic viscosity is associated with oxidation processes at elevated temperatures and a decrease with oil shear or dilution with unburnt fuel. Therefore, a regular replacement of oil forms an essential part of a car maintenance program [1,6]. The assessment of oil viscosity makes it possible to select an appropriate interval between subsequent oil changes, which has a significant impact on the operating and maintenance costs (operation and maintenance) [1].

In recent years, the demand for online oil measurement and data management has been increasing [7]. The sensors are directly installed in the vehicle to constantly monitor the condition of oil. They use different measurement principles depending on the parameter to be monitored, e.g., optical properties (light scattering) or electrical properties (transmittance and conductivity) [8,9]. In terms of viscosity measurements, the following methods are also used: sensing body displacement, acoustic, and vibrational methods [8,10].

Kinematic viscosity is a measure of the resistance to flow of a fluid under the influence of gravity. Determining the kinematic viscosity is based on measuring the flow of the tested liquid through a channel with a given geometry using a device called a capillary viscometer. Determination of viscosity by capillary method is based on Poiseuille’s law and expressed as

$$Q=\frac{\pi \Delta pR^{4}}{8\eta L}$$

where Δ*p*—directly proportional to the pressure difference between the ends of the tube, *R*—radius of the tube, *L*—length of the tube, and *η*—inversely proportional to the viscosity of the fluid.

It consists in measuring the flow time of a given volume of liquid through a calibrated glass capillary with a circular cross-section at a given pressure drop. The kinematic viscosity (ν) is calculated from the formula

ν= Ct 

where C is the viscometer calibration constant, mm^2^/s, and t is the arithmetic mean of the outflow time, s.

Viscosity measurements are important in many industries, as they are a very fast, accurate, and very reliable way to analyze important factors affecting the machine performance [11,12].

The monitoring of oil viscosity consists in determining the changes in viscosity during the operation compared to the viscosity of fresh oil, i.e., the starting value corresponds to the viscosity of the fresh oil, while the critical values for the decrease and increase in viscosity are established [13]. It is, however, worth noting that the viscosity of fresh oil, as given in the standards, may differ from its nominal value by up to 20%, while a change in viscosity of 10% during the operation may often be considered critical [13]. Therefore, fresh oil control is important to obtain a reference value for proper viscosity monitoring [14]. A viscosity assessment usually involves conducting tests in specialized research laboratories that have appropriate equipment. Yet, it is an expensive and logistically complex undertaking, requiring a relatively large sample size and a team of qualified analysts. It also generates large delays between the sampling procedure and the analysis results [15,16,17].

The standard method of measuring the kinematic viscosity (ASTM D445), with the use of different types of capillaries, provides reliable and very precise viscosity results [13]. Yet, thanks to the automation of the measurement processes, the precision of the obtained values may increase further. However, its application requires appropriate laboratory conditions to carry out measurements and time-consuming thermostating, which makes this method unsuitable for determining the viscosity on-site [8,18]. On the other hand, the developed method of the simultaneous determination of dynamic viscosity and density—Stabinger viscometer—can boast many advantages. With this device, quick measurements in a wide temperature range become possible [11]. Unlike the capillary method, the measurement results are obtained in a short time; however, the device is not suitable for field use.

When the machine is in use, decisions often need to be taken based on a quick measurement made on-site without having to face complicated configuration and employing qualified analysts [19]. Of course, enormous efforts have been made to obtain the possibility of making laboratory measurements directly on-site of the machine operation [20]; hence, the further development and growing popularity of devices enabling such practices [8,21]. A good example of devices combining fast measurements, low costs, and high efficiency of the tests on a relatively small sample are the ones that use the FTIR (Fourier-transform infrared spectroscopy) method [22] or micro-channeling. Their compact design makes it possible to actually conduct “field” tests. What is more, such devices usually use databases located in the measuring instrument, created on the basis of known properties of fresh oil samples and permanently serving as a reference point for the analyzed oils of the same type [23,24,25,26,27]. All of these instruments do not require any cumbersome sample preparations and make it possible to obtain the measurement results within a few minutes. Consequently, there is no need to wait for the results of the laboratory analyses, which directly translates into making quick diagnostic decisions related to extending or shortening the time between oil changes. Thus, the costs of operating and using devices can be reduced, and some serious failures may even be prevented [28,29]. However, in order to achieve this, the results obtained using mobile devices (mid-FTIR spectrophotometer and microchannel viscometer) must be comparable with the results obtained using laboratory equipment (capillary and Stabinger viscometer). Hence, the main aim of this paper is to compare the results of kinematic viscosity measurements (obtained with the use of devices enabling a quick viscosity assessment of lubricating oils), with the standardized method requiring the use of Ubbelohde capillaries. Thus, the formulated aim also takes into account the differences between these methods, as described in detail in the methodological section of this paper. To maintain the practical dimension of the study, commercial oils (instead of the reference ones) were used in the tests. The research was carried out on fresh oils only, because used oils contain impurities that could pose additional difficulty for the devices. Notably, used oil samples/or the samples of used oil usually contain significant amounts of soot coming from combustion products. The soot presents some problems on capillaries, because it leads to the formation of deposits on the tube, thus interfering with the fluid flow. This could potentially pose a problem for the microchannel viscometer (in terms of the adequacy of the measurement). Moreover, when it comes to the practicality of such measurements, testing the fresh oil eventually allows to determine changes in the selected parameters of oils during the operation—such samples serve as a reference point, providing the initial viscosity values (in fresh oil). This, in turn, makes it possible to calculate changes in different parameters without seeking references to absolute values.

The correlations of the following methods were analyzed in pairs: Stabinger viscometer–capillary viscometer, mid-FTIR spectrophotometer–capillary viscometer, and microchannel viscometer–capillary viscometer. The outcomes of the comparisons made it possible to assess whether the measurement values obtained with these devices were consistent. Multiple regression was used to assess the consistency of the results. The outcomes may prove to be helpful when making decisions related to the use of a proper measuring instrument.

## 2. Materials and Methods

### 2.1. Materials

The research materials comprised 42 engine oils from various producers. All of the samples tested were synthetic oils of 5W-30 viscosity grade, recommended for passenger cars. The oils were numbered from 1 to 42. The brand names of the engine oils and their quality specifications were not disclosed, as the purpose of the study was to compare the test methods and not to provide a qualitative analysis of the samples tested. The trade names of the devices used in the research were not given either, as the aim was to compare the methods and not the instruments.

In order to compare the selected methods, it was decided to rely solely on fresh oils. Used oils would have to constitute a separate study, since they contain degradation products that may pose additional difficulties in obtaining correct results, especially using the FTIR method. All of the measurements were made in three-fold repetition with each device, and the differences between results (repeatability) could not differ by more than +/−0.5%.

The relative uncertainty value of each oil viscosity was calculated according to references [11,30,31,32]. The expanded uncertainty was calculated by multiplying the relative uncertainty by the coverage factor *k* = 2 to derive the 95% confidence interval. The preparation of the devices and the samples, as well as the measurements, were all carried out in accordance with the instructions found in the user manuals. The kinematic viscosity test methods are presented in Table 1.

All the samples were tested sequentially on the apparatus. During the measurements, the same environmental conditions were maintained, and the samples were thermostatted at 20 °C and mixed before the measurement.

### 2.2. Data Analysis

The obtained results were statistically analyzed using Statistica 13. Multiple regression (MR) was used to assess the compatibility between the measurements obtained with the two methods. It is generally used to analyze the variability of a dependent or criterion variable using information provided by independent or predictor variables [33]. Multiple regression is an important component of the general linear model [34,35,36]. It is also used to compare a given method with another recognized as a standard or to compare the precision of two methods treated equally. In the latter case, it is verified whether both methods are equally accurate. None of them are assumed to be a standard one. By marking the results of the first method as *x* and the second one as *y*, the estimators of linear regression *y* = *b*_0_ + *b*_1_*x* were calculated. Two methods were considered equally accurate if the regression equation took the form of y = *b*_0_ + *x*.

Mahalanobis distances (MD) were used to identify the samples for which it was difficult to obtain convergent results. The following formula was used:

(1) $$MD\left(x\right)=\;\sqrt{{\left(x-\mu \right)}^{T}S^{-1}\left(x-\mu \right)},$$

where *x* is a point, for which the MD was counted from the center of the dataset, *μ* is the middle value of the dataset, and *S* is the covariance matrix of the entire dataset (results obtained with four different methods). To statistically confirm whether one of the devices does not underestimate and the other one does not overestimate the measurement values, a post-hoc signed-rank test with the Bonferroni correction was conducted. The analysis was performed with the use of R software, version 3.6.1. [37].

### 2.3. Test Method

A detailed description of the four methods used in the study is provided below. Some of the basic features of the devices are presented in Table 1 and Table 2.

Method 1. The Capillary Tube Viscometer Test Method (serving as a reference, officially approved)

Kinematic viscosity was determined in accordance with ASTM D445, using the Ubbelohd capillaries (size 2; measurement range 20–100 mm^2^/s and temp. 40 °C).

Method 2. Determination of kinematic viscosity with Stabinger Viscometer

Stabinger viscometer meets ASTM D7042 and D4052 standards. It is intended for measuring the dynamic viscosity and density of liquids, mainly oils, and for converting the obtained results into kinematic viscosity. An innovative solution is an oscillating U-tube measuring the density of the tested sample in the direct vicinity of the viscosity measurement cell. Closing both measuring elements in a well-thermostated chamber, where the temperature is maintained by a cascade Peltier element, and ensuring temperature measurement with a resolution of up to thousandths of a degree Celsius, made it possible to obtain the simultaneous measurements of three parameters: dynamic viscosity, kinematic viscosity, and density at the test temperature.

Method 3. Determination of kinematic viscosity with Microchannel Viscometer

The device is a portable, solvent-free, temperature-controlled kinematic viscometer. It was designed to determine kinematic viscosity at 40 °C in the field for applications where immediate results are essential for assessing the condition of equipment. The Microchannel viscometer has a split cell design that enables the measurement of kinematic viscosity using a 60 µL of oil. When closed, the center pieces of the split cell form a funnel with a 100-micron gap, allowing the oil to flow down by gravity. Sensors along the funnel are triggered when the oil flows by and the flow time between two sensors is measured. The kinematic viscosity is then calculated.

Method 4. Determination of kinematic viscosity with Mid-FTIR Spectrophotometer

This device is a mobile FTIR spectrophotometer. It does not require sample preparation and calibration. The device is preconfigured with preloaded databases containing calibration samples. As a result, the parameters tested can be determined with repeatability and reproducibility in accordance with the requirements of standard ASTM methods. The device has a pump that sucks the sample and introduces it to the measuring system. The Mid-FTIR spectrophotometer uses interferometers, which are the manufacturer’s patented solutions. The device also contains preloaded databases and a multilinear regression to determine the viscosity.

## 3. Results and Discussion

The results of the kinematic viscosity measurements at 40 °C for the oil samples tested are shown in Table 3, where the results, taking into account the expanded uncertainty and the basic descriptive statistics, are presented. Based on the obtained data, it can be concluded that the test results are characterized by a level of variation from several to several dozen percent (coefficient of variation), depending on the device used.

The following oil samples showed the greatest variations in the results: #3, #4, #5, #8, #13, #20, #24, #34, and #36—the standard deviation above 10 mm^2^/s. This is mainly due to the measurement errors; the values obtained with the Mid-FTIR spectrophotometer were generally higher than the “true” ones (except for the sample #20, for which the obtained value was lower). The coefficient of variation for these samples reached the level of 12.8–40.3% (the standard deviation from 10 to 23.8 mm^2^/s).

**Figure 1:**
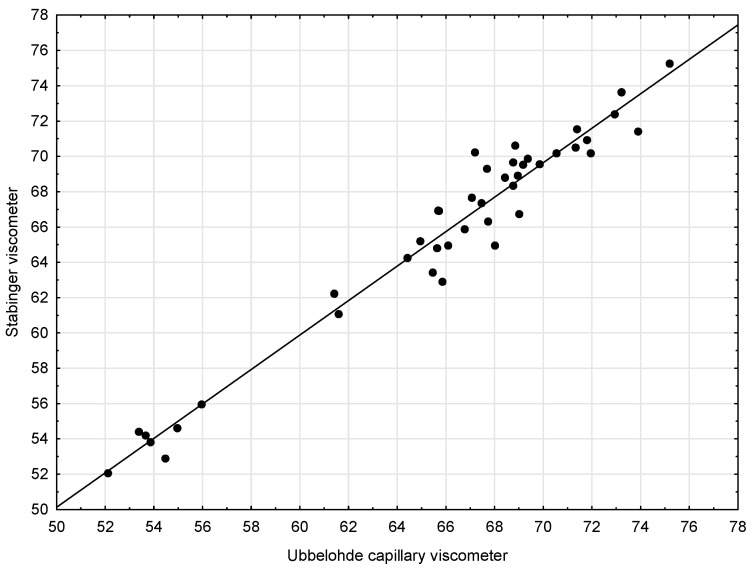
Scatter plots with regression lines for kinematic viscosity—Capillary viscometer and Stabinger viscometer.

In the next step, a scatter chart was elaborated for the compared methods (Figure 1). It has been confirmed that the linear function well-describes the relationship between the results obtained with a Capillary viscometer and Stabinger viscometer.

Then, a linear model was sought. For this purpose, a multiple regression was used. A summary of the regressions is provided below.

The following form of the regression function was obtained:

*y* = 0.9754*x* + 1.3610

It was then checked whether Method 2 (Stabinger viscometer) is as precise as Method 1 (Capillary viscometer) (Table 4). The two sub-hypotheses of the null hypothesis H0: *b*_1_ = 1 and *b*_0_ = 0 were tested simultaneously. Since no grounds for rejecting the null hypothesis were found, this indicates the equivalence of both methods. The hypothesis was verified using the F test defined as follows:

$$F=\frac{nb_{0}^{2}+2n\bar{x}b_{0}\left(b_{1}-1\right)+{\left(b_{1}-1\right)}^{2}x^{2}}{2\sigma_{e}^{2}}$$

where *n*—group size, *b*_0_ and *b*_1_—regression coefficients, $\sigma_{e}^{2}$—residual variance, $\bar{x}$—mean of independent variables *x_i_*, and *x*^2^—expression given by the formula

$$\overset{n}{\underset{i=1}{\sum }}x_{i}^{2}$$

The obtained value was *F* = 1.1780. Consequently, the value of the test probability (*p* = 0.3207) meant that there were no grounds to reject the null hypothesis. In other words, because the null hypothesis was not rejected, statistical confirmation of the similarity of the two methods was obtained, i.e., the following statement was confirmed: The measurements carried out using a Stabinger viscometer are as precise as the measurements conducted using a Capillary viscometer.

**Figure 2:**
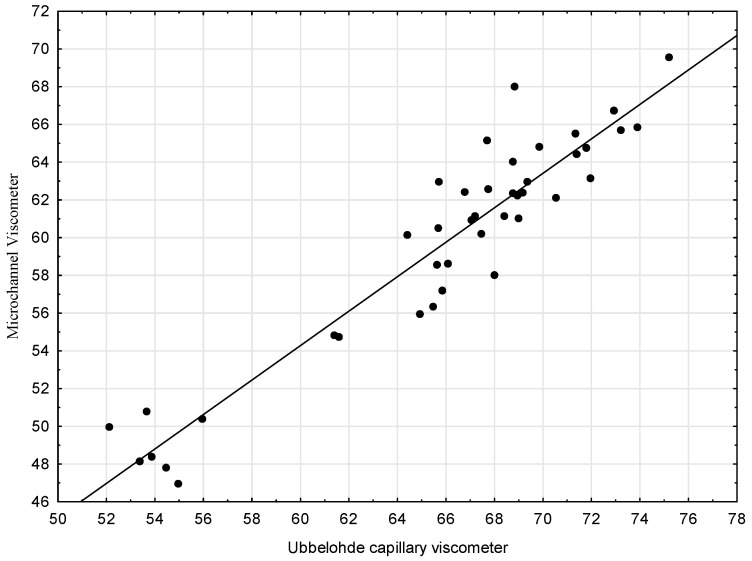
Scatter plots with regression lines for the kinematic viscosity—Capillary viscometer and Microchannel viscometer.

Similar steps were taken to analyze the second pair compared (Capillary viscometer and Microchannel viscometer). Firstly, the scatter charts were prepared (Figure 2). Then, the analysis was performed, and it has been confirmed that the linear function describes the relationship between the variables fairly well (yet worse than in the previously discussed pair).

As a result of the estimation, the following form of the regression function was obtained:

*y* = 0.913442*x* − 0.525197

In this case, it was also checked if Method 3 (Microchannel viscometer) is as precise as Method 1 (Capillary viscometer) (Table 5). Again, the two sub-hypotheses of the null hypothesis H0: *b*_1_ = 1 and *b*_0_ = 0 were tested simultaneously, and the hypothesis was verified using the F test. This time, the value F = 215.164 was obtained. The test probability value (*p* < 0.000001) resulted in the rejection of the null hypothesis. Since the null hypothesis was rejected, it was then checked which of the conditions (sub-hypotheses) was not met. To this end, each sub-hypothesis was verified separately: H0: *b*_1_ = 1 and then Ho: *b*_0_ = 0. For the H_0_ hypothesis *b*_1_ = 1, the value t = 1.73999 was obtained, which meant that there were no grounds for rejecting it (*p* = 0.0896). In turn, for the H_0_ hypothesis *b*_0_ = 0, the value *t* = 0.159354 was obtained, and thus, also, in this case, there were no grounds to reject it (*p* = 0.8742). Overall, the null hypothesis was rejected, even though each of the sub-hypotheses could be accepted separately, which means that Method 3 (Microchannel viscometer) is not as precise as Method 1 (Capillary viscometer) and that the results obtained using it will be subject to the measurement error.

**Figure 3:**
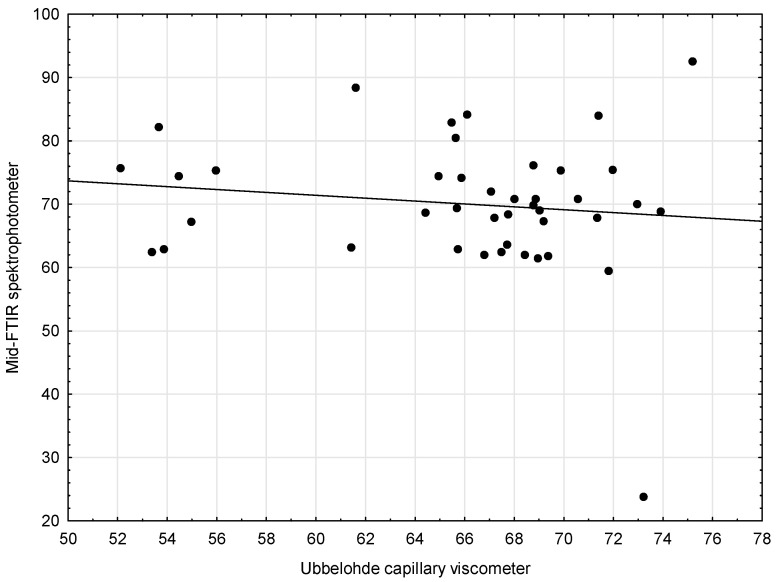
Scatter plots with regression lines for the kinematic viscosity—Capillary viscometer and Mid-FTIR spectrophotometer.

**Figure 4:**
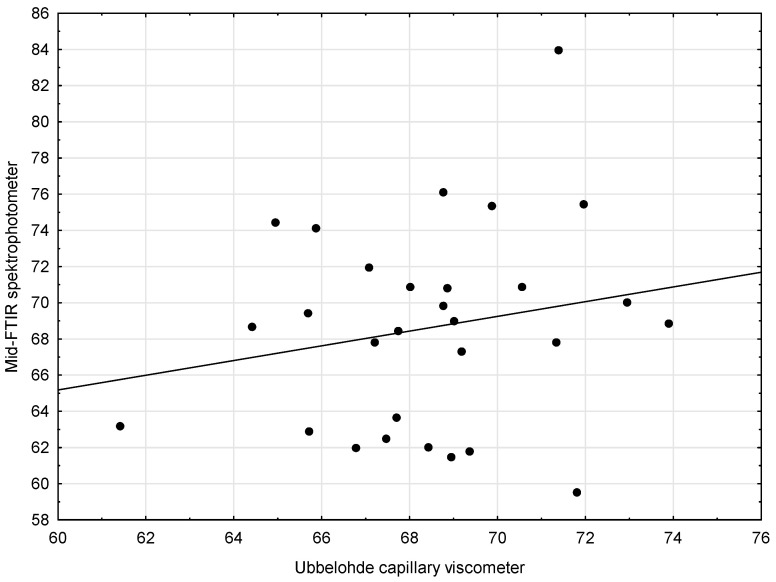
Scatter plots with regression lines for kinematic viscosity—Capillary viscometer and Mid-FTIR spectrophotometer—after removing the outlying samples: #3, #4, #5, #8, #12, #13, #18, #20, #21, #24, #34, #36, and #40.

The third pair of methods (Capillary Viscometer and Mid-FTIR Spectrophotometer) was supposed to be analyzed in a similar way. First, the scatter chart of the compared methods was made. It was then noted that the linear function did not describe the relationship between the variables (Figure 3). When the outlying results were excluded (Figure 4), the situation improved only slightly.

The analysis of the scatter plots of variables for the compared methods showed no linear relationship, which is why there were no grounds to state that both methods could be used interchangeably, giving equally accurate results. The results obtained using Method 4 (Mid-FTIR spectrophotometer) are thus subject to a very large measurement error.

**Figure 5:**
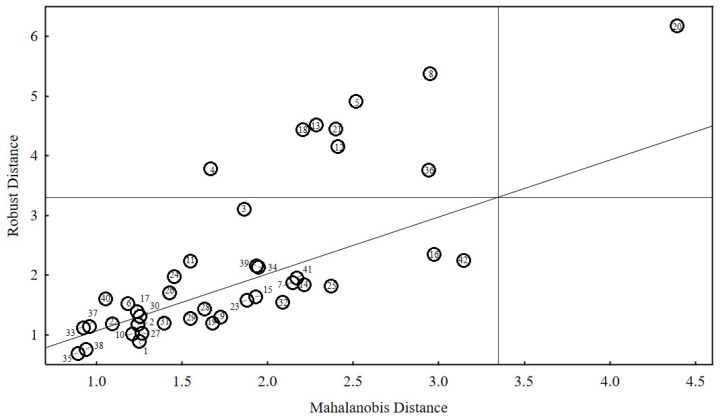
Mahalanobis distance plot.

In the next step, an attempt was made to select outliers to indicate the samples for which the greatest discrepancies in the results obtained using different devices were found. For this purpose, the Mahalanobis distance (MD) was used. A particular advantage of the Mahalanobis distance is that it has a known distribution: it is a chi-square distribution, with the number of degrees of freedom equal to the dimension of the dataset (i.e., the number of features with which the observations are described). Hence, it is assumed that the distances above the 97.5% quantile are outliers. After applying this assumption to the variables studied, one outlier was obtained—the oil sample #20—whereas four other oil samples were close to the “outlying” limit (#8, #36, #6, and #42). Nevertheless, for the statistical method chosen, the assumption that the MD has a chi-square distribution is true only when the data has a multidimensional normal distribution. The collected data did not have a normal distribution. The aim was to search for the outliers, so the distribution, almost by assumption, could not be normal. To solve this problem, the so-called robust covariance matrix estimators were applied. Probably, the most commonly used one is the MCD (Minimum Covariance Determinant). This estimator gave nine outlying oil samples. These were (from the highest to the lowest MD) #20, #8, #5, #13, #21, #18, #12, #36, and #4. The detailed results of the analysis are shown in Figure 5.

The “normal” MD was placed on the horizontal axis. The vertical line around 3.5 is a 97.5% quantile from the chi-square distribution with five degrees of freedom. To the right of it are the outliers mentioned earlier. The robust MD was placed on the vertical axis. The horizontal line is the same quantile (97.5% from the chi-square distribution with five degrees of freedom). It is worth noting that, for example, oil samples #3 and #4 with ordinary MD are placed on the chart around 1.7 and, with robust MD, above 3. In other words, when considering the MD alone, these two samples were very far from outlying, but the robust MD made them either outliers or close to outlying. This means that the transition to robust estimators (i.e., removing the normality assumption) “changed” them into outliers. Therefore, their “outlying” results from the fact that there are connections between the characteristics of oils other than those described by the normal distribution.

The statistical tool used confirmed the observations that were presented in the previous paragraphs. One oil (#20) absolutely stood out from the other oils tested (regardless of the type of MD used). It is an oil for which additional measurements were made using the Mid-FTIR method to confirm the surprisingly low value of kinematic viscosity (24 mm^2^/s), which was obtained in the test. Each subsequent result oscillated around the one obtained earlier. This was most probably due to the limitations of the databases and the method used to estimate the viscosity in the Mid-FTIR spectrophotometer—such a “gap” may apply to this particular oil sample, which as one of very few oils belonging to the three ACEA classes (A3, B3/B4, and C2/C3). According to the API classification, it meets the quality corresponding to the SM/SL and CF classes. The other samples that were classified as outliers (#8, #5, #13, #21, #18, #12, #36, and #4) showed a min. of a 20% higher viscosity (according to the Mid-FTIR spectrophotometer) than the average for the other three methods. The largest difference (after oil sample #20) was found for the samples #8 (47%) and #5 (39%).

**Figure 6:**
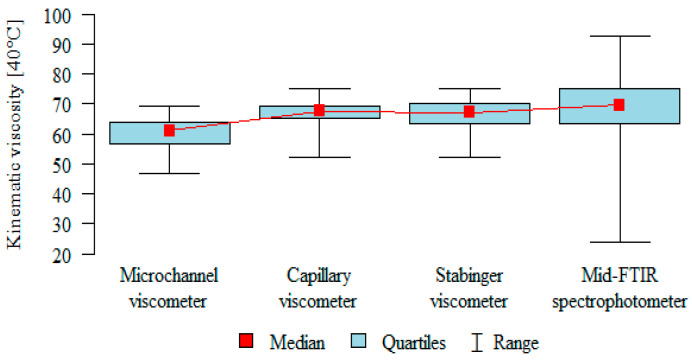
The box plot of the results obtained with the use of the analyzed devices.

Based on previous analyses, it has been noticed that the microchannel viscometer underestimates the results, and the Mid-FTIR spectrophotometer overestimates them compared to the two methods that can be considered homogeneous. To confirm these observations in a statistical manner, a post-hoc analysis was performed (Table 6 and Figure 6). It was found that the results obtained from the microchannel viscometer were significantly lower than the other results (*p* < 0.05). However, no statistical confirmation was obtained for the hypothesis according to which the Mid-FTIR spectrophotometer overestimates the results, because no significant differences were obtained for this method.

**Figure 7:**
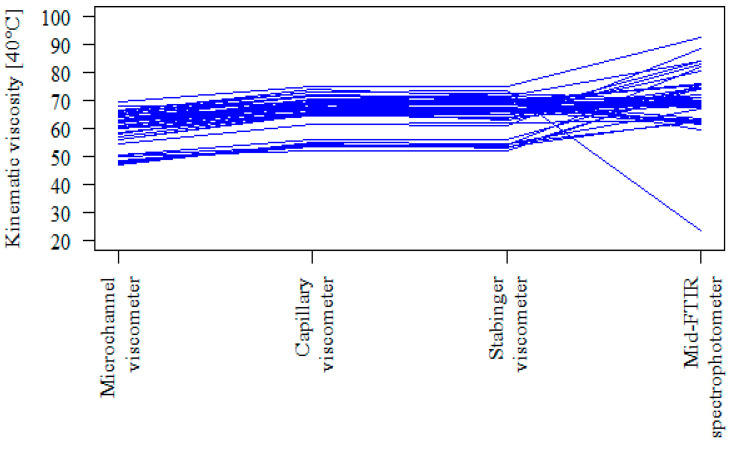
The tracks of the results for individual oil samples.

The above-made observations can be also confirmed by another type of chart (Figure 7). Each line/track in this graph represents one oil sample. For all individual results (42 samples), the microchannel viscometer can be found below the reference methods (Stabinger viscometer and Capillary viscometer). In contrast, for a significant part of the results (15 samples) obtained with the use of the Mid-FTIR spectrophotometer, the lines are at a lower level than for the results obtained using the Capillary viscometer and/or Stabinger viscometer.

## 4. Conclusions

There are many different devices on the market that make it possible to assess the viscosity of a lubricant, and they use various methods of measurement. It is recommended that the selection of such devices is carried out carefully. Some of them facilitate the measurements conducted directly on site, where the machine is operating; yet, the results obtained with them can lead to wrong conclusions, especially when the first measurement is made using the reference method and the subsequent measurements use other methods.

The research experiment carried out showed that the results of the measurements are influenced by the type of research method chosen. Only one of the three analyzed methods (Stabinger viscometer) has a very high compliance with the standardized method and provides as precise results as the reference method. No such confirmation was found for the other two methods. A post-hoc analysis confirmed that the values obtained using the SpectroVisc apparatus are significantly lower than those obtained using the Capillary viscometer and Stabinger viscometer (*p* < 0.05). However, no significant differences were found that could confirm the overestimation of the measurement results obtained with the use of the Mid-FTIR spectrophotometer, which, for a large part of the results (15 samples), showed a great similarity (in terms of accuracy) to the reference methods. Yet, for a vast majority of the results, it failed to provide a correct measurement result. The Mahalanobis distances made it possible to indicate the samples for which the greatest discrepancies were found in the results obtained with the use of different devices. Only nie out of 42 samples showed a min. of a 20% higher viscosity (Mid-FTIR spectrophotometer) than the average for the other three methods. The largest difference (apart from the oil sample #20) was shown for the samples #8 (47%) and #5 (39%).

For as many as nine samples, the standard deviation exceeded 10 mm^2^/s, which should be considered a high value. The coefficient of variation reached 12.8–40.3% for these samples.

Just like with any research studies, especially those empirical in nature, the analysis presented in this article has its limitations. These are mainly conditioned by the method of the sample selection and the sample size. Moreover, in the present study, only fresh oils and only one viscosity grade were examined. Used engine oils would make a separate case of study, because they contain degradation products that may pose additional difficulties in obtaining the correct results, especially when using the FTIR method. It should be noted that three of the examined methods are based on direct measurements, while the mid-FTIR technique is an indirect method for which the viscosity parameters were calculated using MRL (Multiple linear regression) based on the spectra of the oils of known viscosity. The authors deliberately relied on the factory libraries without changing their settings.

An interesting direction of future research may be to focus more attention on creating a separate library for the mid-FTIR spectrophotometer; based on which, the viscosity could be calculated. This could lead to achieving more reliable measurement results. Another possible research direction would be to increase the number of devices tested and to include oils in other viscosity classes. It could also be interesting to verify the reproducibility of the viscosity measurement results for the used oils.

## Figures and Tables

**Table 1:** Basic features of the devices used in the study.

	Capillary Viscometer	Stabinger Viscometer	Mid-FTIR	Microchannel Viscometer
Sample Volume	18 mL	min 3 mL ^1^	approx. 7 mL	0.06 mL
Measurement range Standard Analytical Range @ 40 °C	20–100 mm^2^/s (capillary tube size 2)	0.2–30,000 mm^2^/s	50–100 mm^2^/s	1–700 mm^2^/s
Sample introduction	manually	manual filling with the use of a syringe	automatic, by integrated sample pump	manually
Cleaning	manually	cleaning with the use of a syringe (petrochemical gasoline)	automatic rinsing	manually
Reference measurement	no data	adjusted with 4 standard fluids (oil: L, M, C, H)	automatic, with heptane as reference fluid	no data
Measurement time	min. 240 s	360 s ^2^	60 s	120 s
Calibration	carried out using certified standards			factory calibrated with a matrix of international lubricants
Available Test Methods	ASTM D445 (EN ISO 3104)	ASTM D7042 and D4052	e.g., ASTM D445 (MLR calculations)	ASTM D 8092
Weight	approx. 28 kg	18 kg	approx. 10 kg	1.8 kg
Power supply	mains power supply	mains power supply	mains power supply	built-in rechargeable lithium ion battery

^1^—measurements with supported filling, single measurement with premixing. ^2^—Stabinger Viscometer standard, “precise” class, repeated.

**Table 2:** Measurement repeatability and reproducibility—declared by manufacturers.

	Capillary Viscometer	Stabinger Viscometer	Mid-FTIR	Microchannel Viscometer
Viscosity repeatability	0.26%	0.1% ^2^	≤±3% RSD of value, typical, over range 1–350 mm^2^/s	Repeatability and reproducibility depend on the library used for the MLR calculations
Viscosity reproducibility ^1^	0.76%	0.35% ^2^	≤±3% of measured value over range 1–350 mm^2^/s	

^1^—the difference between two single, independent results, obtained by different analysts working in different laboratories on identical test materials while maintaining the determination procedure described. ^2^—certified for adjustment work by using petroleum-based viscosity standards (does not contain pattern uncertainty).

**Table 3:** Kinematic viscosity measured at 40 °C with the use of four different devices, and the basic descriptive statistics.

Sample Number	Capillary Viscometer	Stabinger Viscometer	Microchannel Viscometer	Mid-FTIR	Average	The Standard Deviation	The Variation	The Coefficient of Variation
	$\bar{x}\;\pm \;U(\nu )\;({\mathrm{mm}}^{2}/s)$							[%]
#1	68.77 ± 0.08	68.33 ± 0.12	64.0 ± 1.9	69.8 ± 0.2	67.7	2.6	6.5	3.8
#2	69.87 ± 0.05	69.55 ± 0.12	64.8 ± 2.2	75.3 ± 0.8	69.9	4.3	18.6	6.2
#3	61.60 ± 0.08	61.06 ± 0.11	54.7 ± 1.9	88.4± 0.2	66.4	14.9	223.4	22.5
#4	55.96 ± 0.10	55.95 ± 0.10	50.4 ± 2.2	75.3 ± 1.2	59.4	10.9	119.6	18.4
#5	53.67 ± 0.10	54.18 ± 0.10	50.8 ± 0.6	82.2 ± 2.4	60.2	14.7	217.0	24.5
#6	64.42 ± 0.13	64.24 ± 0.11	60.1 ± 0.8	68.7 ± 1.2	64.4	3.5	12.1	5.4
#7	73.90 ± 0.03	71.40 ± 0.13	65.9 ± 0.8	68.8 ± 2.2	70.0	3.5	11.9	4.9
#8	52.13 ± 0.05	52.06 ± 0.09	50.0 ± 2.5	75.7 ± 1.8	57.5	12.2	149.3	21.3
#9	67.74 ± 0.08	66.31 ± 0.12	62.6 ± 1.9	68.4 ± 2.4	66.3	2.6	6.9	4.0
#10	65.69 ± 0.08	66.91 ± 0.12	60.5 ± 1.4	69.4 ± 2.0	65.6	3.8	14.1	5.7
#11	61.41 ± 0.10	62.2 ± 0.11	54.8 ± 2.5	63.2 ± 2.2	60.4	3.8	14.4	6.3
#12	54.97 ± 0.03	54.60 ± 0.10	47.0 ± 1.7	67.2 ± 2.6	55.9	8.4	70.3	15.0
#13	54.48 ± 0.03	52.87 ± 0.09	47.8 ± 1.9	74.4 ± 2.4	57.4	11.7	137.3	20.4
#14	64.95 ± 0.13	65.18 ± 0.11	55.9 ± 2.5	74.4 ± 2.0	65.1	7.6	57.1	11.6
#15	66.78 ± 0.13	65.87 ± 0.12	62.4 ± 2.5	62.0 ± 1.6	64.3	2.4	5.9	3.8
#16	68.85 ± 0.08	70.60 ± 0.12	68.0 ± 5.0	70.8 ± 3.2	69.6	1.4	1.8	2.0
#17	72.95 ± 0.13	72.38 ± 0.13	66.7 ± 1.1	70.0 ± 3.0	70.5	2.8	8.0	4.0
#18	53.87 ± 0.13	53.81 ± 0.09	48.4 ± 1.9	62.9 ± 0.2	54.7	6.0	36.0	11.0
#19	69.01 ± 0.10	66.73 ± 0.12	61.0 ± 2.2	69.0 ± 1.4	66.4	3.8	14.3	5.7
#20	73.22 ± 0.13	73.61 ± 0.13	65.7 ± 2.2	23.8 ± 1.6	59.1	23.8	565.9	40.3
#21	53.4 ± 0.15	54.39 ± 0.10	48.1 ± 3.3	62.4 ± 1.0	54.6	5.9	34.8	10.8
#22	69.37 ± 0.13	69.86 ± 0.12	63.0 ± 2.5	61.8 ± 1.2	66.0	4.2	17.7	6.4
#23	65.72 ± 0.13	66.91 ± 0.12	63.0 ± 2.2	62.9± 1.8	64.6	2.0	4.1	3.1
#24	66.1 ± 0.13	64.94 ± 0.11	58.6 ± 0.6	84.2 ± 1.6	68.5	11.0	120.8	16.1
#25	68.02 ± 0.05	64.94 ± 0.11	58.0 ± 1.9	70.9 ± 2.4	65.5	5.5	30.4	8.4
#26	71.81 ± 0.08	70.92 ± 0.12	64.8 ± 2.5	59.5 ± 0.8	66.7	5.8	33.2	8.6
#27	71.34 ± 0.10	70.50 ± 0.12	65.5 ± 2.8	67.8 ± 0.6	68.8	2.7	7.0	3.9
#28	71.96 ± 0.13	70.17 ± 0.12	63.1 ± 0.6	75.4 ± 1.6	70.2	5.2	26.8	7.4
#29	68.77 ± 0.10	69.65 ± 0.12	62.3 ± 1.7	76.1 ± 1.8	69.2	5.6	31.8	8.1
#30	68.43 ± 0.03	68.79 ± 0.12	61.1 ± 2.2	62.0 ± 3.0	65.1	4.1	16.7	6.3
#31	70.55 ± 0.08	70.17 ± 0.12	62.1 ± 1.9	70.9 ± 0.2	68.4	4.2	17.8	6.2
#32	67.70 ± 0.10	69.29 ± 0.12	65.2 ± 6.1	63.7 ± 1.4	66.4	2.5	6.4	3.8
#33	68.96 ± 0.13	68.89 ± 0.12	62.2 ± 2.8	61.5 ± 0.4	65.4	4.1	16.8	6.3
#34	65.47 ± 0.05	63.42 ± 0.11	56.3 ± 2.5	82.9 ± 1.2	67.0	11.3	127.8	16.9
#35	67.08 ± 0.05	67.65 ± 0.12	60.9 ± 2.2	72.0 ± 0.8	66.9	4.5	20.6	6.8
#36	75.20 ± 0.08	75.25 ± 0.13	69.5 ± 0.8	92.6 ± 1.0	78.1	10.0	99.7	12.8
#37	67.47 ± 0.08	67.34 ± 0.12	60.2 ± 2.8	62.5 ± 2.2	64.4	3.6	13.2	5.6
#38	69.18 ± 0.08	69.52 ± 0.12	62.4 ± 1.9	67.3 ± 0.8	67.1	3.3	10.8	4.9
#39	71.40 ± 0.08	71.53 ± 0.13	64.4 ± 3.3	83.9 ± 1.8	72.8	8.1	66.1	11.2
#40	65.64 ± 0.05	64.80 ± 0.11	58.6 ± 1.9	80.5 ± 0.6	67.4	9.3	86.3	13.8
#41	65.87 ± 0.03	62.89 ± 0.11	57.2 ± 2.2	74.1 ± 0.8	65.0	7.1	49.8	10.9
#42	67.21 ± 0.08	70.20 ± 0.12	61.1 ± 2.5	67.8 ± 0.2	66.6	3.9	14.9	5.8

**Table 4:** The estimation results for the measurements carried out with a Stabinger viscometer and Capillary viscometer.

	*b*’	the Standard Error (*b*’)	*b*	the Standard Error (*b*)	*t*(40)	*p*
An intercept parameter			1.36	2.15	0.63	0.53
Capillary Viscometer	0.98	0.03	0.98	0.03	30.11	0.00

*b*’—the coefficient of determination, *b*—the coefficients of linear regression.

**Table 5:** The estimation results for the measurements carried out with the Microchannel viscometer and Capillary viscometer.

	*b*’	The Standard Error (*b*’)	*b*	The Standard Error (*b*)	*t*(40)	*p*
An intercept parameter			−0.53	3.30	−0.16	0.87
Capillary Viscometer	0.95	0.05	0.91	0.05	18.36	0.00

*b*’—the coefficient of determination, *b*—the coefficients of linear regression.

**Table 6:** The mean values ($\bar{x})$, standard deviations (SD), and *p* *-values.

	Microchannel Viscometer (*N* = 42)—A	Capillary Viscometer (*N* = 42)—B	Stabinger Viscometer (*N* = 42)—C	Mid-FTIR (*N* = 42)—D	*p* *
$\bar{x}\;\pm \;SD$	59.74 ± 5.93	65.97 ± 6.14	65.71 ± 6.12	70.06 ± 10.74	*p* < 0.001
median	61.13	67.59	67.12	69.62	
quartiles	56.54–63.8	65.08–69.32	63.62–70.09	63.29–75.34	D, B, C > A

* If, for at least one measurement, the distribution is not normal, the Friedman test + post-hoc analysis results (Wilcoxon Signed-Rank test with the Bonferroni correction).

## Data Availability

The data presented in this study are available on request from the corresponding author.
